# Antibiotic Utilization and Resistance According to the WHO AWaRe Classification in Intensive Care Units After COVID-19 Third Wave in Pakistan: Findings and Implications

**DOI:** 10.3390/medicina61030481

**Published:** 2025-03-10

**Authors:** Muhammad Shahid Iqbal, Mohd Faiyaz Khan, Sadaf Farooqui, Salah-Ud-Din Khan, Saeed Vohra, Shahzad Rasheed, Muhammad Zahid Iqbal, Shafqat Qamer

**Affiliations:** 1 Department of Clinical Pharmacy, College of Pharmacy, Prince Sattam bin Abdulaziz University, Al-Kharj 11942, Saudi Arabia; 2 Department of Biochemistry, College of Medicine, Imam Mohammad Ibn Saud Islamic University (IMSIU), Riyadh 11432, Saudi Arabia; 3 Department of Anatomy and Physiology, College of Medicine, Imam Mohammad Ibn Saud Islamic University (IMSIU), Riyadh 11432, Saudi Arabia; 4 Department of Clinical Pharmacy, College of Pharmacy, King Khalid University, Abha 61421, Saudi Arabia; 5 Department of Pharmacy Practice, Faculty of Pharmaceutical Sciences, Lahore University of Biological & Applied Sciences, Lahore 53400, Pakistan; 6 Department of Basic Medical Sciences, College of Medicine, Prince Sattam bin Abdulaziz University, Al-Kharj 11942, Saudi Arabia

**Keywords:** antibiotics, AMR, COVID-19, ICUs, AWaRe, AMS, WHO, Pakistan

## Abstract

*Background and Objective:* Irrational use and overuse of antibiotics is considered a major cause of antimicrobial resistance (AMR) among patients admitted to hospitals, especially in intensive care units (ICUs). ICUs are the most critical wards in healthcare settings, where the use of antibiotics is much higher compared to other wards. Therefore, the appropriate administration and monitoring of antibiotic usage in these units is a matter of concern. *Materials and Methods:* This retrospective study evaluated the types, utilization patterns, sensitivity, and resistance of various antibiotics used among patients admitted to the ICUs of different hospitals after the third wave of the coronavirus disease in 2019 (COVID-19) in Pakistan. *Results:* It was observed that more than 40% of the patients were given two antibiotics and 54.3% were given at least one antibiotic each day. A total of 768 antibiotics from different groups, based on the World Health Organization (WHO) Access, Watch, and Reserve (AWaRe) classification, were prescribed to 313 patients admitted to ICUs between April and August 2021. Among the types of antibiotics, amoxicillin/clavulanic acid was the most frequently used antibiotic (75 prescriptions). It was also observed that the majority of the bacterial isolates were more sensitive to carbapenems than the other antibiotics. The current study showed that antibiotic usage according to the AWaRe classifications was 31.8% in the Access category, 59.5% in the Watch category, and 8.7% in the Reserve category in ICUs of the studied hospitals after the third wave of COVID-19. *Conclusions:* The findings of the study also highlight the importance and continuous need for the implementation of antibiotic stewardship programs to optimize the appropriate utilization of antibiotics in the ICUs of the studied hospitals.

## 1. Introduction

Antimicrobial resistance (AMR) has been partially managed after the introduction of the World Health Organization (WHO) Access, Watch, and Reserve (AWaRe) classification of antibiotic usage [1]. The WHO Expert Committee on Selection and Use of Essential Medicines developed the AWaRe tool to lessen the clinical burden, address the global spread of AMR, and minimize the side effects associated with irrational antibiotic use [1,2,3,4]. The three categories of the AWaRe classification are as follows: “Access” (the first- and second-choice antibiotics, such as amoxicillin, ampicillin, and amikacin, with a narrow spectrum and less potential for resistance), “Watch” (broader-spectrum antibiotics such as fluoroquinolones, macrolides, and second- and third-generation cephalosporins, carrying a higher risk of developing resistance), and Reserve (the last-resort choice antibiotics to treat infections, such as linezolid and colistin, resistant to multiple drugs) [4,5,6]. The WHO AWaRe classification system has two major objectives. The first is reporting antibiotic use according to AWaRe classification worldwide. The second objective is setting targets and assessing and implementing the outcome of antimicrobial stewardship (AMS) initiatives that aim to reduce AMR and maximize apt antibiotic usage [2,3,4,5,6]. AMS, a program that encourages prudent antibiotic use and precise prescribing patterns of antibiotics, also includes the AWaRe tool [7,8]. Various countries like Brazil, the UK, Bangladesh, and Germany have adopted this AWaRe classification tool and found it beneficial in controlling AMR [2,9]. Adoption of the AWaRe classification can also be utilized to track antimicrobial usage trends and encourage sensible prescription patterns among the prescribers [10,11,12]. By encouraging the sensible use of antibiotics in healthcare settings, AMS initiatives significantly contribute to the reduction of AMR [13].

Recent WHO initiatives have highlighted the necessity of structured AMS approaches to address issues brought on by the Coronavirus disease 2019 (COVID-19) pandemic, which resulted in a rise in antibiotic use (despite fewer bacterial co-infections) in a post-pandemic era [14]. Though its implementations are still lacking, WHO’s AWaRe categorization has been progressively included in AMS frames worldwide to help with appropriate antibiotic selection [15]. Furthermore, to implement precise antibiotic usage, AMS programs in low- and middle-income countries often face budget constraints and resource restrictions [16]. This necessitates the need for continuous evaluations of AMS programs all over the world, particularly in low-income countries, to enhance their effectiveness and their alignment with WHO guidelines.

Multidrug-resistant microorganisms have been documented in various parts of Pakistan in the past few years. In Pakistan, Enterobacteriaceae resistance to quinolones has increased over the past 10 years [17,18]. The Salmonella outbreaks in 2016 were resistant to fluoroquinolones [18,19]. According to a prior study, 94% of bloodstream bacteria were resistant to third-generation cephalosporins [19,20]. Several other studies have also reported that various factors contribute enormously to AMR. These include irrational antibiotic prescriptions, incorrect and under-dosing prescribing patterns, antibiotic self-medication, incompetent healthcare staff, insufficient self-training, lack of culture and sensitivity (C&S) testing, and discontinuation of AMS programs [20,21].

In Pakistan, pharmaceutical companies can aggressively market and sell antibiotics without adequate quality control due to the lack of strict monitoring and lenient regulations of the Drug Regulatory Authority of Pakistan (DRAP) [22,23,24]. Another factor contributing to AMR in Pakistan is overprescription and self-medication among the majority of the public [22,23,24]. In Pakistan, many prescribers prescribe antibiotics needlessly, while others do so in response to patient requests [23,24]. Furthermore, a lot of people buy antibiotics from drug stores and pharmacies without any legitimate prescription, making self-medication widespread, which is another contributing factor for AMR in Pakistan [24,25,26]. Furthermore, broad-spectrum antibiotics are frequently prescribed in Pakistan rather than narrow-spectrum antibiotics, which further aids in the development of AMR [24,25]. The general public’s lack of awareness and inadequate education regarding the apt usage of antibiotics is another significant factor contributing to AMR in Pakistan [22,23,24]. Typically, pharmaceutical companies also provide prescribers with hefty incentives to over-prescribe their products, which also results in AMR [24,25]. Some studies have reported that the prescription rate of irrational antibiotics in Pakistan is quite high, i.e., >50% as compared to other developing countries [22,26]. Due to the lack of precise and updated AMS programs in hospitals, abundant irrational antibiotics are being prescribed with less or no effect against most of the microbes [27]. This is concerning because, to enhance WHO AWaRe recommendation adherence and minimize AMR, the susceptibility patterns of microorganisms should be closely observed at primary, secondary, and tertiary care facilities across the entire country [18,19,24].

Intensive care units (ICUs) are the most sensitive areas in hospitals where critically ill patients are kept under intensive care. In ICUs, irrationality and overuse of antibiotics not only result in AMR but can also reduce antibiotic effectiveness [28]. As bacteria become resistant to antibiotics, providing therapy for various life-threatening infections like sepsis, CNS infections, and intra-abdominal infections becomes more challenging in ICUs [27,28]. AMR often leads to longer hospital stays, higher healthcare costs, decreased therapeutic outcomes, and ultimately an increased mortality rate [27,28,29]. Antibiotic use in ICUs is around ten times higher than in the other wards of the hospitals [28,29].

ICUs are critical settings in hospitals where broad-spectrum antibiotics are frequently prescribed and used due to the high prevalence of severe infections [30]. However, inappropriate and unnecessary antibiotic prescription and utilization often results in AMR, making treatments more complex and prolonged in the ICUs [30,31]. According to a recent study, around 50% of antibiotics prescribed in ICUs are unnecessary and inappropriate [30,31,32]. Therefore, monitoring antibiotic prescription is crucial to identify concerning signs of misuse or overuse in ICUs [31,32,33]. Many low and middle-income countries have struggled to implement a robust antimicrobial stewardship program (ASP), especially as recommended by the WHO. Unfortunately, more than half of hospitals in Pakistan do not have any ASP that could help to combat AMR.

In January 2021, the third COVID-19 wave was officially declared by the WHO after a new variant of SARS-CoV-2 (B.1.1.7) from the United Kingdom was detected in more than 70 countries including Pakistan [34,35,36]. This variant was also associated with an increased risk of casualties compared to previous variants. In Pakistan, an average of more than 100 patient deaths was reported daily due to this variant [35,36]. Major cities in Pakistan like Islamabad, Lahore, Faisalabad, Multan, and Peshawar were affected the most by B.1.1.7 during the third wave of COVID-19. A strict lockdown was imposed by the Government of Pakistan in these cities to control the spread of the third wave of COVID-19 [36].

Various studies reported that during the COVID-19 pandemic, several infected patients were admitted to hospitals for inappropriate antimicrobial therapy, which resulted in increased AMR worldwide [37,38]. Conversely, there were several reasons to prescribe antibiotics during the COVID-19 pandemic, especially to treat COVID-19 and non-COVID-19 patients suffering from different bacterial infections. These infections included pneumonia, co-infections, secondary bacterial infections, and several nosocomial infections [39]. According to another study, a large number of hospitalized patients were given empirical antimicrobial therapies during the COVID-19 pandemic which was not appropriate at all and led to an increase in AMR [38]. Some other studies also reported that during the COVID-19 pandemic, a higher number of patients admitted to ICUs were administered unnecessary antibiotics [37,38,39,40]. Overall, the frequency of various infections increased dramatically during the third wave of COVID-19 across the globe [41]. Therefore, this study was designed to determine types, utilization (according to AWaRe classification), sensitivity, and resistance patterns of various antibiotics used in patients admitted to the ICUs of different hospitals in Pakistan. This was a pioneering study, as no previous research has been conducted in the ICUs of the studied hospitals during or after the COVID-19 third wave.

## 2. Materials and Methods

### 2.1. Study Design and Study Period

A retrospective study was conducted between April and August 2021 in the ICUs of four public hospitals in Pakistan. All aspects of the study protocol, including information about participants’ identity and background, were strictly confidential and for research purposes only. This study was approved by the Research Ethics Committee, Lahore Pharmacy College, Lahore Medical & Dental College under ETH/LPC/06/01/21.

### 2.2. Data Management and Confidentiality

All subject names were kept in a password-protected folder and were allocated with study identification numbers. Identification numbers instead of patient names were used in the final dataset of this research. On completion of the study, data from the computer were copied to a USB flash drive, and computer data were deleted permanently. As per the international research data storage requirements, a USB flash drive and any hardcopy data were only stored by the investigators and kept for a minimum of three years from the completion of the study. No one other than investigators were allowed to view the study data.

### 2.3. Inclusion and Exclusion Criteria

The study inclusion criteria were patients aged 18 to 60, suffering from any infection, admitted to the ICUs during the study period, and treated with at least one antibiotic. Patients admitted to other wards/departments of the hospitals were excluded from the study. All of the patients who were admitted to ICUs during the study period were strictly assessed to meet the eligibility criteria. All patients meeting the inclusion criteria were included in the study. The study sample (N) of our study was 313, with *n* = 82 in hospital A, *n* = 91 in hospital B, *n* = 76 in hospital C, and *n* = 64 in hospital D.

### 2.4. Study Setting and Data Collection

All the selected hospitals are categorized as public hospitals in Pakistan. All these hospitals have the capacity of, on average, 800–1000 beds. The average number of beds in the ICUs for infectious diseases is around 30–40 beds in these hospitals. The data were collected from the patient files/records assigned to each patient in the hospitals. The records were further verified by staff nurses. The records of antibiotic treatment of the patients were followed up until their discharge from the ICUs. The number of patients discharged on special request, shifted to the general ward, and shifted to specialized wards were also recorded and included among the outcomes of the antibiotic therapy provided. The demographic details (age and gender), reason for admission, and length of stay were also recorded. The severity of illnesses and physiological and clinical characteristics of ICU-admitted patients were determined using the revised Acute Physiology and Chronic Health Evaluation II (APACHE II) score and Glasgow Coma Scale. Antibiotics were classified using the Anatomical Therapeutic Chemical (ATC) Classification System and their utilization was presented in the form of a daily-defined dose (DDD) per 100 bed days as presented in another study [42]. Further evaluation was made using the type of antibiotic therapy (empirical, definitive, prophylactic), number of antibiotics given, and route of administration. The sensitivity and resistance data were collected from each patient’s file/record (which the hospital obtained from the microbiology tests performed and recorded in individual files). Antibiotics were classified using the WHO AWaRe classification system.

### 2.5. Statistical Analyses

The data were entered in Microsoft Excel version 2023 and analyzed using Statistical Package for the Social Sciences (SPSS) ver. 25. The obtained data were analyzed using Kolmogorov–Smirnov test to confirm the normal distribution of numeric variables. Univariate analyses comprising Chi-square test and Fisher’s exact test were used to determine the association between demographic variables and antibiotic usage. Comparisons of categorical variables were also made where appropriate using Chi-square test and Fisher’s exact test. A multivariate analyses comprising multinomial logistic regression was also performed to adjust the effects of potential demographic and clinical factors (predictors) that were significant in univariate analyses (*p* < 0.05).

## 3. Results

Table 1 describes the demographic and clinical characteristics of patients across four selected hospitals. The main demographic and clinical characteristics include age distribution, gender, reasons for admission, length of hospital stay, and therapy outcomes. Total patients included in the study were 313, including *n* = 82 from hospital A, *n* = 91 from hospital B, *n* = 76 from hospital C, and *n* = 64 from hospital D. The number and percentage of males were 28 (34.1%), 38 (41.4%), 29 (38.2%), and 21 (32.8%), while the females were 54 (65.9%), 53 (64.6%), 47 (61.8), and 43 (67.2%) in hospitals A, B, C, and D, respectively. The highest number of patients (*n* = 92, 29.39% of the total) suffered from respiratory tract infection followed by renal infection (*n* = 82, 26.19%). The length of stay in ICUs ranged from 1 to >7 days. The majority of the patients (>50%) stayed for 2–3 days. Our results also showed that 33 (40.2%) patients were shifted to the general ward in hospital A, 36 (39.6%) in hospital B, 31 (40.8%) in hospital C, and 24 (37.5%) in hospital D. Moreover, for hospitals A, B, C, and D, the total number of patients discharged on request were 28 (34.2%), 33 (36.3%), 24 (31.6%), and 22 (34.4%), respectively. The APACHE (II) score of more than 50% of the patients was 11–20. Statistically significant differences were observed for length of stay (days) (*p* < 0.001) and therapy outcomes (*p* = 0.036).

According to the obtained results, a statistically significant difference in length of stay (*p* < 0.001) was observed among all four hospitals, with hospital C having a longer patient stay. Additionally, a statistically significant association (*p* = 0.036) was also found in the therapy outcome variable, with hospital A reporting a higher number of deaths.

Total antibiotic consumption based on the DDD(g) and DDD per 100 bed days during the study period was 54.2. Antibiotic types, their consumption based on the DDD/100-bed days, ATC codes, and the number of prescriptions is shown in Table 2.

Furthermore, it was also observed that hospital D followed more appropriate antibiotic prescribing patterns, with prescribers generally adhering to WHO AWaRe classification, i.e., prescribing fewer antibiotics from the Reserve group. However, the percentage of antibiotic usage from the Watch group was similar among all four of the studied hospitals. Antibiotic types, their consumption in each hospital, and the number (percentage) of prescriptions are shown in Table 3.

Our study findings showed a statistically significant difference (*p* < 0.001) in the utilization of Watch category antibiotics, indicating variation in prescription patterns across hospitals. The highest consumption of Watch antibiotics was reported in hospital B and hospital C, suggesting differences in ICU antibiotic stewardship policies. Additionally, the use of Reserve antibiotics was relatively low in all hospitals, reflecting restricted usage for last-resort antibiotics.

Among antibiotic therapy types, the majority of patients were on definitive therapy in all four hospitals, with 34 (41.5%), 37 (40.6%), 34 (44.7%), and 27 (42.2%) patients on definitive therapy in hospitals A, B, C, and D, respectively. The total number of patients on empirical therapy were 29 (35.4%), 33 (36.3%), 24 (31.6%), and 24 (37.5%) and as prophylaxis were 19 (23.1%), 21 (23.1%), 18 (23.7%) and 13 (20.3%) in hospitals A, B, C, and D, respectively. More than 40% of the total studied patients were treated with two antibiotics and the route of administration in the majority of the cases (>50%) was intravenous. These results are presented in Table 4.

A statistically significant association (<0.001) in the number of antibiotics used per day was observed among the studied hospitals. In hospital B, the highest number of antibiotics used was two (41.8% of patients), while in hospital D, the highest number was also two (40.6% of patients).

According to the results of the C&S test of hospital D, isolates of gram-positive bacteria showed highest sensitivity to meropenem (100%), followed by imipenem/cilastatin (75%), ceftazidime (75%), piperacillin/tazobactam (66.7%), amikacin (66.7%), and lincomycin (60%). Gram-negative bacteria in hospital D showed the highest sensitivity to imipenem/cilastatin (75%), meropenem (66.6%), amikacin (60%), and azithromycin (56.3%) among the total prescriptions sent for C&S analysis. These results are presented in Table 5 and Table 6.

In univariate analyses, among the studied demographic and clinical characteristics variables, only gender, length of stay (days), number of antibiotics given per day, and route of administration were found to be statistically significant (*p* < 0.05) and showed statistically significant differences in AWaRe classification-based usage of antibiotics. These statistically significant variables (predictors) were further analyzed in multivariate analyses using multinomial logistic regression to find pure predictors of antibiotic usage according to the WHO AWaRe categorization among the patients studied. Table 7 presents the multinomial logistic regression analyses results which revealed that for any change in length of stay (days), i.e., >1 day, the scores were 0.197, 0.276, 0.569, and 0.914, for 2–3 days, 4–5 days, 6–7 days, and >7 days, respectively, in relation to the Reserve category antibiotics usage (*p* < 0.05), when adjusted for other variables. Similarly, a change in the number of antibiotics given per day, i.e., one or more antibiotics, was associated with an increase of 0.219, 0.787, and 1.293 for two antibiotics, three antibiotics, and four or more antibiotics, respectively, in the Reserve category (*p* < 0.05), when other demographic and clinical variables kept constant. The variables of gender and route of administration were not statistically significant (*p* > 0.05).

## 4. Discussion

### 4.1. Demographics and Clinical Characteristics

The global consumption of antibiotics has reached 65% in the last decade and the major cause behind this is the irrational use of antibiotics, especially in developing countries including Pakistan. The reason behind this could be that antibiotics are usually considered a relatively fast remedy to treat infections by numerous prescribers, especially fresh graduates from developing countries [42]. The current study aimed to determine the type, utilization pattern, sensitivity, and resistance pattern of antibiotics used in ICUs of four different hospitals after the third wave of COVID-19 in Pakistan.

In the current study, out of 313 patients, more than 35% were 18 to 35 years old and more than 60% were females. The majority of the patients in ICUs suffered from respiratory tract infections, i.e., they were 31.7%, 29.7%, 27.6%, and 28.1% in hospitals A, B, C, and D, respectively. The presence of renal infections was 24.4%, 26.4%, 27.6%, and 26.6% in hospitals A, B, C, and D, respectively. The other infections include CNS infections, renal, UTIs, and miscellaneous. Length of stay for the maximum number of patients was 2–3 days, i.e., 51.2%, 49.5%, 49.9%, and 53.1% for hospitals A, B, C, and D, respectively. According to the results of this study, there was no statistically significant association observed between some of the studied patients’ demographics and the hospital types, i.e., hospital A, B, C, and hospital D. For instance, in age, gender, and reason for admission variables, there were statistically non-significant associations observed (*p* = 0.301, *p* = 0.122, and *p* = 0.097, respectively). In all four studied hospitals, to control all possible confounders and ensure the similarity between them, all of the demographic characteristics of the patients were matched to obtain the actual usage pattern of the antibiotics among patients admitted to the ICUs.

In the literature, it is reported that most of the prescribers consider the use of broad-spectrum antibiotics as empirical therapy which is similar to our study findings. In the current study, it was found that 54.3% of patients were provided with at least one DDD of antibiotics each day. This was higher than the DDD found in the study conducted by Marasine et al. (2021) in ICUs in Nepal [42]. It was found that the DDD/100 bed days for the four most frequently prescribed antibiotics were 7.6 (amoxicillin/clavulanic acid), 4 (cefotaxime), 1.9 (ampicillin), and 2 (ceftriaxone). In another study [43] by Saleem et al. (2021), it was observed that co-amoxiclav, ciprofloxacin, azithromycin, and levofloxacin were the four most commonly dispensed antibiotics in community pharmacies in Pakistan with DDD percentages of 24.3%, 11.6%, 6%, and 8.2%, respectively. The increased DDDs can be attributed to the fact that in Pakistan most physicians prescribe antibiotics based on the assumption of increased AMR among the public due to their irrational overuse.

There were statistically significant differences observed between some of the clinical characteristics of the patients and the hospital types, i.e., hospital A, B, C, and hospital D. For example, regarding the length of stay (days), the association was statistically significant (*p* < 0.001), which showed that hospital D employed better antibiotic prescription practices (prescribing antibiotics appropriately) as per the WHO AWaRe classification. It is worth mentioning that there was also a statistically significant (*p* < 0.001) relationship observed between the type of the hospital and length of stay (days) among the patients of the studied hospitals. We found that in hospital D, where more appropriate use of antibiotics was observed, the length of stay for patients in ICUs was less than in the rest of the hospitals. A statistically significant association (*p* = 0.036) was observed between the therapy outcomes and the studied hospitals, with hospital D showing better therapy outcomes compared to hospitals A, B, and C. These included deaths, discharge on request, and shifting to the wards from ICUs. These study results corroborate the findings of other studies where a higher rate of antibiotic consumption and prolonged hospital stays were observed [42,43,44]. There could be many reasons/factors that caused non-adherence to practice guidelines (WHO or local) among the prescribers in hospitals A, B, and C. These factors include limited resources, where healthcare systems may not be able to implement prescribing guidelines effectively, lack of awareness where providers may be unaware of or unfamiliar with WHO guidelines, and inadequate training where providers may not receive adequate training, i.e., Continuing Medical Education (CME) about AMR, and AMS [45,46]. In hospital D, according to the hospital practice manual for physicians, all of the prescribers were highly encouraged by the top management to attend at least two CMEs annually, which could be another reason for their better adherence to WHO practice guidelines.

It was observed that in hospital D, the conditions of the patients improved rapidly and were shifted to general wards more frequently compared to the rest of the hospitals. From our study findings, it was also observed that in hospital D, patients’ length of stay in ICUs was shorter, and shifting to the general wards from the ICUs (indicating improved recovery) was more frequent compared to the rest of the hospitals. Comparatively, a better prescription pattern was observed among prescribers in hospital D, which may be attributed to their strong adherence to the WHO practice guidelines. However, in our results, some prescribers tend to prioritize their own experience over the guidelines. This justification is further supported by a recent study (2024) conducted by Dobrowolski et al., which suggested that certain prescribers rely on their own perceptions of patients, diseases, and treatment plans over the recommended WHO guidelines when evaluated for reasons of non-adherence to practice guidelines [47]. Other potential reasons may include clinical factors like complicated cases, patients with multiple comorbidities, local resistance patterns where prescribers may adjust prescriptions based on local antibiotic resistance patterns, and patient-specific factors where physicians may consider allergies or organ dysfunction while prescribing antibiotics to their patients [48,49].

### 4.2. Antibiotic Types and Utilization

The most prescribed antibiotics were amoxicillin/clavulanic acid (*n* = 75) followed by metronidazole (*n* = 66), cefotaxime (*n* = 60), ampicillin (*n* = 59), ceftriaxone (*n* = 57), cefuroxime (*n* = 48), and imipenem (*n* = 48). The higher rate of antibiotic prescription could be due to a lack of precise and relevant knowledge about antibiotics among physicians and non-compliance among patients. A study in Punjab, Pakistan, regarding antibiotic usage concluded that 60.2% of participants did not have appropriate knowledge about antibiotics utilization, and 59.6% of participants were in favor of using antibiotics for self-medications [46]. AMR is a leading healthcare concern that often results in decreased efficacy and increased treatment expenditures [48,49,50]. In another study conducted in Nepal by Marasine et al. (2021), it was found that piperacillin was the most frequently used antibiotic because of an increased prevalence of respiratory infections in ICUs, which is also parallel to the results found in the current study [42]. In a study conducted by Sozen et al. (2013) in Turkey [51], cephalosporins were found to constitute about 57% of all antibiotics used in the hospitals followed by fluoroquinolones (14.7%) and penicillin (10%), which is also in line with the findings of our study.

Lack of diagnostic resources, non-compliance with the WHO AWaRe classification usage, and less information about antibiotic sensitivity patterns are among the major problems of AMR that Pakistan currently facing in general [14,15]. This makes it harder to prescribe antibiotics according to the WHO AWaRe recommendation, which could lead to a further increase in AMR [18,19,40]. According to our study results, hospital B was found to be less adherent to WHO AWaRe recommendations, as there was more frequent usage of Watch category antibiotics when compared to the rest of the hospitals. The reason could be that practicing physicians considered it more convenient and timesaving to prescribe antibiotics, irrespective of AMR issues in the country [47,48,49,50,51,52]. These results corroborate the findings of other studies where the highest rate of antibiotic consumption was observed [41,42,43]. These results are deeply concerning in light of worries about Pakistan’s rising AMR threshold, an increasing rate of irrational antibiotic prescription trends without C&S testing, and the lack of national antibiotic prescribing guidelines at present [22,23,24].

It was noted that the majority of patients were treated with definitive antibiotic therapies and were prescribed more than two or more antibiotics. Dohou et al. (2022) reported that 57% of patients in ICUs were prescribed two to five antibiotics [53]. Their study findings are parallel to the findings of the current study. It has also been reported that antibiotics are often used as prophylaxis for various diseases in many countries [53,54,55]. The results of this study can be compared with those from other countries, though some differences may exist due to geographical variations. In Western Europe, 71% of patients in ICUs were observed to have received various antibiotics as prophylaxis for various illnesses, which is similar to the findings of this study [54,55]. Recently, a study conducted in Belgium found that 42% of the studied patients were prescribed prophylactic antibiotic therapy [54]. Patient-related factors like patient expectations, where prescribers may prescribe antibiotics due to patient expectations or pressure, and lack of patient education, where patients may not understand the risks and benefits of antibiotics, can also lead to overprescription [53,54,55,56,57,58,59]. Some cultural factors, such as patient beliefs and values, can also influence prescribers’ antibiotic prescribing practices [57,58,59,60]. Our study results are also in line with another study conducted in Japan where it was noted that 34% of patients in ICUs were prescribed antibiotics for prophylaxis [56]. Regarding empirical and prophylactic usage of antibiotics among patients admitted in ICUs, the utilization of antibiotics was greater in hospital A compared to hospital D. In Pakistan, empirical and definitive antibiotics therapies are more commonly prescribed, similar to what is reported in our study [57,61]. These findings were similar to other findings, where their perception of the rational use of antibiotics was also poor [59,60,61].

Undeniably, AMR is a major concern regarding high morbidity, mortality, and increased treatment costs worldwide. However, it is particularly important to address AMR in developing nations like the Asia-Pacific region. Antibiotic-resistant microorganisms are thought to be abundant in third-world countries like Pakistan. AMR is predicted to rise in the Asia-Pacific region by 70%, posing a global and local threat [57,58,59]. Globally, South Asian developing nations like Pakistan pose a serious healthcare concern due to increasing AMR. Numerous initiatives from the WHO like AWaRe classification and the importance of AMS are ongoing, but, unfortunately, the actual implementation of AMS in developing countries like Pakistan has not been initiated or completed so far.

There were statistically significant differences observed among the studied hospitals and their usage of antibiotics in terms of AWaRe classification. According to the obtained results, Access category antibiotics usage in ICUs was almost double (41.7%) in hospital D than in hospitals A (28%) and B (21.7%). The difference was also statistically significant (*p* < 0.001). In the Watch category, all of the ICUs in the studied hospitals used almost similar numbers of antibiotics, i.e., hospitals A (60.6%), B (66.4%), C (55%), and D (54%). In terms of the Reserve category, hospitals A and B used antibiotics almost 2–3 times more i.e., 11.4% and 11.9%, respectively, than hospital D (3.8%). The difference was also statistically significant (*p* < 0.001). It can be said that hospital D was more cautious in using antibiotics from the Reserve category in the ICU. Various miscellaneous factors like time constraints, where prescribers may not have time to thoroughly evaluate patients and follow guidelines, defensive prescribing where prescribers may prescribe antibiotics to avoid potential lawsuits or criticism, and the lack of accountability where prescribers may not be held accountable for their prescribing habits can significantly contribute towards irrational antibiotic prescribing practices.

Similarly, it can also be said that patients’ self-preferences were not entertained in hospital D as compared to the hospital B and prescribers were strict about prescribing the antibiotics. For instance, in therapy outcomes, discharge on the request was less in hospital D as compared to the other hospitals like hospitals A and B. Hospital D was also found to be better than the rest of the hospitals, as the length of the stay in ICUs among the admitted patients was less (*p* < 0.001) than the hospital B, C and A. According to the obtained results, there was a statistically significant association observed (*p* = 0.039) between antibiotic therapy-type usage and the studied hospitals. Likewise, in the number of antibiotics given per day, the association was statistically significant (*p* < 0.001) between the studied hospitals, which also showed that hospital D was somehow adherent to WHO AWaRe classification compared to the rest of the hospitals. In terms of route of administration of antibiotics, a statistically significant association (*p* = 0.021) was observed whereby hospital A used more parenteral administrations than the other hospitals.

According to the WHO 30th General Program of Work 2021, a country-level target of a maximum of 60% of total antibiotics consumption should be from the Access category. This means that Access antibiotics should be used more widely than the other 2 categories as per the WHO recommendations, but not more than 60%. The Access category lists the recommended antibiotics for the top 25 infections [4,62]. These antibiotics have to be always accessible, reasonably priced, and of guaranteed quality. The Watch category comprises the majority of the “highest-priority and critically important antibiotics” that should be used to control/treat particular infections that the Access category fails to control. Watch antibiotics should only be used in certain, and restricted situations. Another reason could be that the results of the Watch group antibiotics are better in efficacy than Access group, but they are generally expensive, broad-spectrum, and are more prone to be resistant. Therefore, the WHO recommends their usage to be reduced and only for particular cases, to avoid potential resistance. The Reserve category contains the antibiotics that the WHO recommends only be used as a last resort. These antibiotics ought to be reserved for extreme situations in which all other options have been exhausted. Watch and Reserve categories are nine to 32 times more expensive, respectively, than the Access category antibiotics [4,62].

### 4.3. Antibiotic Sensitivity and Resistance

This study showed that gram-positive bacteria were more sensitive to amikacin, azithromycin, meropenem, and imipenem/cilastatin. Furthermore, it was also inferred that gram-positive bacteria were resistant to vancomycin, ceftazidime, ciprofloxacin, and cefuroxime. Regarding gram-negative bacteria, this study showed that gram-negative bacteria were more sensitive to azithromycin, colistin, lincomycin, and cefotaxime. Furthermore, it was also inferred that gram-negative bacteria were resistant to amoxicillin/clavulanic acid, ampicillin, ciprofloxacin, and cefuroxime. It was also observed that carbapenems were among the most sensitive antibiotics against all bacterial isolates.

Not only physicians but also the general public play an important role in the increase of AMR [63,64]. In line with our study results, similar findings were reported in a study done in Nepal, where gram-negative bacteria were found to be highly resistant to amoxicillin/clavulanic acid, ampicillin, ciprofloxacin, and cefuroxime [65]. Similarly, they also found that gram-negative bacteria were most sensitive to azithromycin, colistin, lincomycin, and cefotaxime, which is also observed in our study [66]. Similar to our study’s findings, two other studies also reported that carbapenems were among the most sensitive antibiotics against all bacterial isolates [47,66].

In Pakistan, another paramount factor that profoundly contributes to decreased sensitivity and increased AMR is the easy access of the general public to antibiotics at drug stores and pharmacies. Sadly, it is a common practice that various types of antibiotics can be accessed and purchased without a prescription from a registered medical practitioner [65,66,67]. Moreover, customer service in the majority of medicine stores and pharmacies in Pakistan is also untrained and non-professional, leading to an increased practice of inappropriate sales of antibiotics which ultimately results in increased AMR [63,64,65,66,67]. According to a recent study (2020), more than 97% of pharmacies and medicine stores in Pakistan contribute enormously to AMR by giving/providing antibiotics without a valid medical prescription. This malpractice plays a significant role in furthering the irrational use and overuse of antibiotics and promoting antibiotic self-medication [57]. A study reported that more than 70% of antibiotic prescriptions were for minor ailments and some were even for viral infections [61]. Several other studies also reported that in Pakistan, more than 70% of the antibiotics were dispensed without valid prescriptions [57,61,68].

In Pakistan, resistance to some antibiotics like ceftriaxone and quinolone, which are often used to treat non-typhoidal Salmonellae, is also rising [64,65,66,67]. Another study reported that around 70% of AMR was present against cefaclor, novobiocin, amoxicillin, and ampicillin [69]. In addition, multidrug resistance (MDR) is also on the rise in Pakistan [69,70,71]. According to another study, nearly 78% of the screened isolates were resistant to at least three antibiotics [70]. A study conducted in Pakistan also reported an overall increase in AMR in different areas of Pakistan, including the appearance of MDR [71]. This increased AMR strongly supports the impression that the Pakistani nation is experiencing a sharp rise in AMR or is perhaps at the advent of MDR [69,70,71]. Another study also reported that >80% of various Escherichia coli isolates were resistant to beta-lactam antibiotics in Lahore, Pakistan [70]. Many of these isolates were also resistant to trimethoprim-sulfamethoxazole and fluoroquinolones [69,70,71]. Overall, our study findings underscore the urgent need for the implementation of AMS programs in all healthcare facilities. Our findings also emphasize strictly adhering to WHO AWaRe classifications of antibiotics usage to ensure appropriate and optimal use of antibiotics in all healthcare facilities across Pakistan, especially in ICUs, to combat AMR.

The daunting problem of AMR in Pakistan can be significantly reduced with the support of strict government policies at the federal, provincial, and local levels, by implementing a code of conduct for the prescribers regarding the prescription of antibiotics according to WHO AWaRe classification, and by arranging CMEs and allied refresher courses like AMS programs. Priority must be given right away to strategies and initiatives that emphasize the responsible use of antimicrobials and restrict their unwanted and irrational use in all healthcare settings. It is anticipated that these actions will significantly lower current infection rates, alter resistance patterns, lower treatment costs, and enhance therapeutic outcomes. In addition to these regulations, the AMR threat can also be mitigated by enhancing public awareness about infectious diseases, providing pure (hygienic) drinking water, and establishing infection control units/centers at the grassroots level in Pakistan.

### 4.4. Factors Influencing AWaRe Classification-Based Usage of Antibiotics

In our study, pure predictors of AWaRe classification-based use of antibiotics were identified by adjusting or controlling confounders using a multinomial logistic regression model. In multinomial logistic regression analyses, it was inferred that in the Reserve category, the ‘length of stay (days)’ variable for 2–3 days (AOR 0.197; *p* = 0.035), 4–5 days (AOR 0.276; *p* = 0.029), 6–7 days (AOR 0.569; *p* = 0.047), and >7 days (AOR 0.914; *p* = 0.041) was statistically significant when 1 day was considered as ‘referent’. Likewise, in the Reserve category, the ‘number of antibiotics given per day’ variable for two antibiotics (AOR 0.219; *p* = 0.014), three antibiotics (AOR 0.787; *p* = 0.032), and four or more antibiotics (AOR 1.293; *p* = 0.048) was statistically significant when one antibiotic use was considered as ‘referent’. These findings affirmed that in the Reserve category, length of stay (days) and number of antibiotics given per day were the pure predictors of AWaRe classification-based usage of antibiotics among the studied patients when adjusted for the other demographic and clinical characteristics variables/confounders. Both of the predictors showed a statistically significant positive association in the Reserve category with AWaRe classification-based usage of antibiotics among the studied patients in ICUs.

In our study, for any change in length of stay (days), i.e., 2–3 days, 4–5 days, 6–7 days, and >7 days, the odds of usage of antibiotics in the Reserve category were increased to 0.197, 0.276, 0.569, and 0.914, respectively. Our results are in accordance with another study, conducted in Nepal, where it was reported that usage of antibiotics was significantly increased with the increase in the length of the hospital stay (days) [42]. Another study conducted in Sweden also presented similar findings regarding length of the hospital stays [72]. Our results in terms of hospital stay (days) also corroborate with the findings of another study that reported prolonged usage of antibiotics with prolonged hospital stays [73]. Regarding the number of antibiotics given per day, our study findings are in line with studies conducted by Marasine et al. [42] and Walther et al. [72], where it was observed that whenever the Reserve category was more frequently used in ICUs, the total number of antibiotics administrated significantly increased. Similar findings to our study results were also reported by another study in terms of the total number of antibiotics administered per day [73]. Our study results indicated that the odds of Reserve category antibiotics usage in ICUs significantly increased for longer hospital stays and simultaneous administration (overuse) of more than two antibiotics. Multivariate analyses indicated that in the study sample, the usage of Reserve category antibiotics increased with the increase in length of stay (days) and increase in number of antibiotics per day.

The inappropriate utilization of antibiotics in ICUs in Pakistan further endangers human lives since uncontrolled increasing bacterial infections have a considerably high negative impact on human health [29,72,73]. Implementing the AWaRe classification system in hospitals in Pakistan, especially in ICUs, can have numerous benefits. It can enable prescribers to make informed decisions about antibiotic prescription, reducing the misuse of broad-spectrum antibiotics. Additionally, by promoting the Access category (narrow-spectrum, first-line options), hospitals can minimize the risk of AMR and reduce the risk of adverse reactions, like Clostridioides difficile infections, allergic reactions, and antibiotic-associated diarrhea. This, in turn, will enhance patient safety, improve health outcomes, and slow the development of AMR. Moreover, by reserving broad-spectrum antibiotics (Watch and Reserve categories) for specific situations, hospitals can also help preserve antibiotics’ effectiveness, reducing the risk of antibiotic failure and improving patient outcomes. Implementing the AWaRe classification system can also lead to reduced costs and shorter hospital stays, as patients are more likely to receive effective, targeted antibiotic therapy, reducing the need for prolonged hospitalization. The AWaRe classification system can further help hospitals manage MDR organisms more effectively, reducing the risk of transmission and improving the overall health-related quality of life of patients.

Therefore, there is a greater need for the development of strict policies as well as their implementation regarding the appropriate and precise utilization of antibiotics in Pakistan. In Pakistan, prescribers predominantly face challenges when prescribing antibiotics due to a lack of AMS programs, developing healthcare systems, and a dearth of diagnostic facilities [29,69,70,71,72,73]. The results of the study could be used not only to evaluate the current healthcare situation in ICUs in some hospitals of Pakistan but would also help to design, implement, and standardize policies and practices regarding safe use of antibiotics, especially according to the WHO AWaRe classification. This necessitates the need to adapt AMS programs similar to those of developed nations. Our study recommends the Ministry of Health, Pakistan, to instruct and enforce all the healthcare facilities to design and implement continuous AMS programs and instruct all prescribers to attend. Authorities can also declare that such programs will be part of their CMEs and will be compulsory to attend for their practice license renewals. This will ultimately promote and encourage prescribers to strictly adhere to the rational use of antibiotics according to WHO AWaRe classification.

### 4.5. Strengths and Limitations of the Study

Strengths of the study include that it can help understand the prevalence of antibiotics utilization patterns, sensitivity, and resistance in ICUs of four different hospitals after the third wave of COVID-19 in Pakistan. This study’s results would also aid in identifying and defining suitable AMS programs for proper antibiotic utilization to combat AMR in hospitals, especially in ICUs in Pakistan. The study results would also help in developing awareness among people about antibiotic irrational use and its consequences in the form of increased AMR.

The current study has a few limitations. Firstly, data was collected from the patients’ records/files, which may not be very accurate, and results may be affected due to imprecise accuracy of the records (incomplete data or missing data) and prone to selection bias as compared to prospective studies. Second, this type of study could be prone to misclassification bias. Third, retrospective studies could also be subject to unavoidable variables, i.e., some other related factors may also be present that were ignored or not evaluated. Fourth, increasing the sample size or study sample can further improve the outcome of the study, though we tried to increase it by making a comparative study of four different hospitals. Fifth, the study results cannot be perfectly generalized for overall antibiotic consumption in all provinces of Pakistan since only four hospitals were included in this study. Sixth, our results do not exactly reflect the availability and the cost of the antibiotics used in different hospitals, as cheaper antibiotics are more frequently available and used than expensive antibiotics in third-world countries like Pakistan. In this study, some of the patients were already on antibiotics when they were admitted to a hospital, and the hospital was required to complete that particular antibiotic course, which could mislead the results, making it unfair to accuse a particular hospital. In the future, drug utilization studies (DUS) should be conducted to determine the exact level of adherence of prescribers to the local, regional, or WHO antibiotics’ prescribing guidelines.

## 5. Conclusions

This study clearly shows that in managing various types of infections (even if they are of an analog origin), physicians or medical practitioners, regardless of their experience and education, often choose different antibiotics, which further results in increased hospitalizations and complications among patients suffering from various infections. This strongly necessitates the adoption of national, regional, or WHO protocols for antibiotic utilization in an apt way. There is also a need to strengthen awareness and education programs on proper antibiotic use among healthcare providers, patients, and the general public. More studies are needed in Pakistan to strengthen the apt usage of antibiotics, especially according to the WHO guidelines, particularly the AWaRe classification. In response to the growing AMR crisis, the implementation of AMS has become increasingly imperative as AMS strongly advocates for the sensible use of antibiotics, optimizing treatment outcomes, and minimizing the development of AMR. Additionally, implementing AMS programs in ICUs can also help optimize antibiotic use, monitor prescribing practices, and promote adherence to the WHO guidelines and protocols.

The majority of the studied hospitals in Pakistan are not fully following the WHO Committee of 2019 recommendations or the WHO 30th General Program of Work 2021 regarding apt antibiotic usage, which should be followed and practiced. Prescribers should adopt WHO guidelines, especially the AWaRe classification, to ensure the rational use of antibiotics in Pakistan and to be in line with the WHO initiatives for implementing AMS programs for antibiotics worldwide. In fact, some hospitals in Pakistan have already taken initiatives to make it compulsory for prescribers to strictly adhere to the WHO AWaRe classification and to use antibiotics accordingly to avoid increasing trends of AMR in Pakistan.

## Figures and Tables

**Table 1:** Demographic and clinical characteristics of patients (*n* = 313).

Characteristics	Categories	Hospitals								*p*-Value
		Hospital A (*n* = 82)		Hospital B (*n* = 91)		Hospital C (*n* = 76)		Hospital D (*n* = 64)		
		*n*	%	*n*	%	*n*	%	*n*	%	
Age	<18	17	20.7	18	19.7	21	27.6	11	17.2	0.301 ^a^
	18–35	31	37.8	43	47.3	18	23.7	24	37.5	
	36–55	27	32.9	26	28.6	31	40.8	16	25	
	>55	7	8.6	4	4.4	6	7.9	13	20.3	
Gender	Male	28	34.1	38	41.4	29	38.2	21	32.8	0.122 ^a^
	Female	54	65.9	53	64.6	47	61.8	43	67.2	
Reason for admission	Respiratory tract infection	26	31.7	27	29.7	21	27.6	18	28.1	0.097 ^a^
	Renal infection	20	24.4	24	26.4	21	27.6	17	26.6	
	CNS infection	16	19.5	17	18.7	14	18.4	12	18.8	
	GIT infection	9	11.0	11	12.1	9	11.8	8	12.5	
	Urinary tract infection	8	11.7	8	8.8	9	11.8	6	9.4	
	Miscellaneous	3	3.7	4	4.4	2	2.6	3	4.7	
Length of stay (days)	1	20	24.4	21	23.1	16	21.1	12	18.8	<0.001 ^b^
	2–3	42	51.2	45	49.5	38	49.9	34	53.1	
	4–5	12	14.6	15	16.5	13	17.1	9	14	
	6–7	3	3.7	4	4.4	4	5.3	5	7.8	
	>7	5	6.1	6	6.5	5	6.6	4	6.3	
Therapy outcomes	Death	5	6.1	4	4.3	4	5.2	3	4.7	0.036 ^b^
	Discharged on request	28	34.2	33	36.3	24	31.6	22	34.4	
	Shifted to general ward	33	40.2	36	39.6	31	40.8	24	37.5	
	Shifted to specialized ward	16	19.5	18	19.8	17	22.4	15	23.4	
APACHE(II) score	0–10	19	23.3	22	24.5	17	22.6	14	22.1	0.059 ^a^
	11–20	48	59.3	51	56.3	44	57.8	38	59.4	
	21–30	12	14.2	13	13.4	12	15.8	9	13.8	
	31–40	3	3.2	5	5.8	3	3.8	3	4.7	
	3–5	4	4.9	5	5.5	6	7.9	6	9.4	0.053 ^a^
GCS Score	6–8	15	18.3	19	20.9	15	19.7	10	15.6	
	9–12	42	51.2	43	47.3	36	47.4	27	42.2	
	13–15	21	25.6	24	26.4	19	25.0	21	32.8	

^a^ Chi-square test; ᵇ Fisher’s exact test: *n* = total number of patients included in the study from each hospital. APACHE: Acute Physiology and Chronic Health Evaluation; GCS: Glasgow Coma Scale. Hospital C had a longer patient stay (*p* < 0.001); Hospital A had a higher number of deaths (*p* = 0.036).

**Table 2:** Antibiotic types and their utilization in ICUs of the studied hospitals in DDD(g).

	Types	ATC Code	No. of Prescriptions	%	DDD (g)	ACI (DDD/Bed Days × 100)
Access	Amoxicillin/clavulanic acid	J01CR02	75	9.8	7.6	4.5
	Metronidazole	J01XD01	66	8.6	2.1	4.2
	Ampicillin	J01CA01	59	7.7	1.9	3.1
	Amikacin	J01GB06	44	5.6	9.9	7.5
Watch	Cefoperazone	J01DB09	21	2.7	2	2.8
	Ceftazidime	J01DB05	20	2.6	2	2.6
	Ciprofloxacin	J01MA02	38	4.9	1	1.3
	Cefotaxime	J01DD01	60	7.8	4	2.3
	Azithromycin	J01FA10	37	4.8	0.3	1.1
	Lincomycin	J01FF02	29	3.8	1.8	4.1
	Meropenem	J01DH02	29	3.8	2	2.3
	Cefuroxime	J01DC02	48	6.3	0.5	1.8
	Vancomycin	J01XA01	25	3.3	1.9	2.2
	Imipenem/cilastatin	J01DH51	48	6.3	2	1.5
	Ceftriaxone	J01DD04	57	7.4	2	1.6
	Piperacillin/tazobactam	J01CR05	45	5.9	14	8.3
Reserve	Linezolid	J01XX08	38	4.9	1.2	2.3
	Colistin	J01XB01	29	3.8	9 (MU)	0.7
	Total antibiotic consumption		768	100		54.2

**Table 3:** Antibiotic type and their utilization in ICUs of the studied hospitals.

AWaRe Classification 2021	Types	Hospital A	No. of Prescriptions (%)	Hospital B	No. of Prescriptions (%)	Hospital C	No. of Prescriptions (%)	Hospital D	No. of Prescriptions (%)	*p*-Value
Access	Amoxicillin/clavulanic acid	17	49 (28)	14	49 (21.7)	20	68 (37.8)	24	78 (41.7)	<0.001 ^b^
	Metronidazole	13		13		19		21		
	Ampicillin	11		12		17		19		
	Amikacin	8		10		12		14		
Watch	Cefoperazone	4	106 (60.6)	9	150 (66.4)	5	99 (55)	3	102 (54.5)	
	Ceftazidime	4		9		4		3		
	Ciprofloxacin	9		17		7		5		
	Cefotaxime	13		26		10		11		
	Azithromycin	8		10		9		10		
	Lincomycin	7		9		8		5		
	Meropenem	6		4		9		10		
	Cefuroxime	10		12		12		14		
	Vancomycin	5		9		6		5		
	Imipenem/cilastatin	14		15		9		10		
	Ceftriaxone	12		20		11		14		
	Piperacillin/tazobactam	14		10		9		12		
Reserve	Linezolid	11	20 (11.4)	18	27 (11.9)	6	13 (7.2)	3	7 (3.8)	
	Colistin	9		9		7		4		
	Total antibiotic consumption	768	175 (100)		226 (100)		180 (100)		187 (100)	

^b^ Fisher’s exact test: Hospital B and Hospital C consumed more “Watch” antibiotics.

**Table 4:** Antibiotic administration.

Characteristics	Categories	Hospitals								*p*-Value
		Hospital A (*n* = 82)		Hospital B (*n* = 91)		Hospital C (*n* = 76)		Hospital D (*n* = 64)		
		*n*	%	*n*	%	*n*	%	*n*	%	
Antibiotic therapy type	Empirical	29	35.4	33	36.3	24	31.6	24	37.5	0.039 ^b^
	Definitive (after C&S test)	34	41.5	37	40.6	34	44.7	27	42.2	
	Prophylaxis	19	23.1	21	23.1	18	23.7	13	20.3	
Number of antibiotics given per day	1	17	20.7	19	20.9	14	18.4	14	21.9	<0.001 ^b^
	2	35	42.7	38	41.8	35	46.1	26	40.6	
	3	16	19.5	15	16.5	16	21.1	14	21.9	
	≥4	14	17.1	19	20.9	11	14.5	10	15.6	
Route of administration	Parenteral (intravenous)	67	81.7	74	81.3	57	75	53	82.8	0.021 ^b^
	Oral	15	18.3	17	18.7	19	25	11	17.2	

^b^ Fisher’s exact test: *n* = total number of patients included in the study from each hospital.

**Table 5:** Antibiotic sensitivity and resistance against gram-positive bacteria.

AWaRe Classification 2021	Types		Gram-Positive (Sensitivity)					Gram-Positive (Resistance)				
		*n*	N_1_	Hospital A	Hospital B	Hospital C	Hospital D	N_2_	Hospital A	Hospital B	Hospital C	Hospital D
Access	Amoxicillin/clavulanic acid			*n* (%)	*n* (%)	*n* (%)	*n* (%)		*n* (%)	*n* (%)	*n* (%)	*n* (%)
		17	13	4 (30.7)	3 (23.1)	3 (23.1)	3 (23.1)	4	1 (25)	1 (25)	1 (25)	1 (25)
	Metronidazole	5	4	1 (25)	3 (75)	0 (0)	0 (0)	1	0 (0)	1 (100)	0 (0)	0 (0)
	Ampicillin	11	9	1 (11.1)	5 (55.6)	2 (22.2)	1 (11.1)	2	1 (50)	1 (50)	0 (0)	0 (0)
	Amikacin	4	3	1 (33.3)	0 (0)	0 (0)	2 (66.7)	1	0 (0)	0 (0)	1 (100)	0 (0)
Watch	Cefoperazone	9	8	2 (25)	1 (12.5)	2 (25)	3 (37.5)	1	1 (100)	0 (0)	0 (0)	0 (0)
	Ceftazidime	7	4	0 (0)	0 (0)	1 (25)	3 (75)	3	1 (33.3)	0 (0)	1 (33.4)	1 (33.3)
	Ciprofloxacin	5	1	0 (0)	1 (100)	0 (0)	0 (0)	4	1 (25)	3 (75)	0 (0)	0 (0)
	Cefotaxime	3	2	0 (0)	0 (0)	1 (50)	1 (50)	1	1 (100)	0 (0)	0 (0)	0 (0)
	Azithromycin	9	7	1 (14.3)	1 (14.3)	1 (14.3)	4 (57.1)	2	0 (0)	0 (0)	1 (50)	1 (50)
	Lincomycin	5	5	0 (0)	1 (20)	1 (20)	3 (60)	0	0 (0)	0 (0)	0 (0)	0 (0)
	Meropenem	4	4	0 (0)	0 (0)	0 (0)	4 (100)	0	0 (0)	0 (0)	0 (0)	0 (0)
	Cefuroxime	12	9	2 (22.2)	4 (44.5)	1 (11.1)	2 (22.2)	3	1 (33.3)	1 (33.3)	0 (0)	1 (33.4)
	Vancomycin	7	6	1 (16.7)	1 (16.7)	1 (16.6)	3 (50)	1	0 (0)	0 (0)	0 (0)	1 (100)
	Imipenem/cilastatin	8	8	1 (12.5)	0 (0)	1 (12.5)	6 (75)	0	0 (0)	0 (0)	0 (0)	0 (0)
	Ceftriaxone	22	14	3 (21.4)	4 (28.6)	3 (21.4)	4 (28.6)	8	1 (12.5)	2 (25)	2 (25)	3 (37.5)
	Piperacillin/tazobactam	4	3	0 (0)	0 (0)	1 (33.3)	2 (66.7)	1	0 (0)	0 (0)	0 (0)	1 (100)
Reserve	Linezolid	11	9	3 (33.3)	0 (0)	3 (33.3)	3 (33.4)	2	0 (0)	0 (0)	1 (50)	1 (50)
	Colistin	4	2	0 (0)	0 (0)	1 (50)	1 (50)	2	1 (50)	0 (0)	0 (0)	1 (50)

N_1_ = prescriptions where gram-positive (sensitivity) was checked; N_2_ = prescriptions where gram-positive (resistance) was checked. *n* = total prescriptions where gram-positive (sensitivity) and gram-positive (resistance) were checked; NP = not performed.

**Table 6:** Antibiotic sensitivity and resistance against gram-negative bacteria.

AWaRe Classification 2021	Types		Gram-Negative (Sensitivity)					Gram-Negative (Resistance)				
		*n*	N_1_	Hospital A	Hospital B	Hospital C	Hospital D	N_2_	Hospital A	Hospital B	Hospital C	Hospital D
Access	Amoxicillin/clavulanic acid			*n* (%)	*n* (%)	*n* (%)	*n* (%)		*n* (%)	*n* (%)	*n* (%)	*n* (%)
		32	14	3 (21.4)	4 (28.6)	3 (21.4)	4 (28.6)	18	4 (22.2)	5 (27.8)	5 (27.8)	4 (22.2)
	Metronidazole	6	2	0 (0)	2 (100)	0 (0)	0 (0)	4	0 (0)	2 (50)	0 (0)	2 (50)
	Ampicillin	9	6	1 (16.7)	3 (50)	1 (16.7)	1 (16.6)	3	1 (33.4)	1 (33.3)	1 (33.3)	0 (0)
	Amikacin	14	10	2 (20)	0 (0)	2 (20)	6 (60)	4	0 (0)	0 (0)	2 (50)	2 (50)
Watch	Cefoperazone	10	9	2 (22.2)	3 (33.4)	2 (22.2)	2 (22.2)	1	1 (100)	0 (0)	0 (0)	0 (0)
	Ceftazidime	8	6	1 (16.7)	1 (16.7)	1 (16.6)	3 (50)	2	0 (0)	0 (0)	1 (50)	1 (50)
	Ciprofloxacin	11	8	1 (12.5)	5 (62.5)	1 (12.5)	1 (12.5)	3	1 (33.3)	2 (66.7)	0 (0)	0 (0)
	Cefotaxime	10	8	2 (25)	2 (25)	2 (25)	2 (25)	2	0 (0)	0 (0)	1 (50)	1 (50)
	Azithromycin	17	16	2 (12.5)	2 (12.5)	3 (18.7)	9 (56.3)	1	0 (0)	0 (0)	1 (100)	0 (0)
	Lincomycin	11	4	0 (0)	0 (0)	2 (50)	2 (50)	7	1 (14.3)	1 (14.3)	2 (28.6)	3 (42.8)
	Meropenem	7	6	1 (16.7)	0 (0)	1 (16.7)	4 (66.6)	1	0 (0)	0 (0)	0 (0)	1 (100)
	Cefuroxime	9	6	1 (16.7)	2 (33.3)	2 (33.3)	1 (16.7)	3	1 (33.4)	1 (33.3)	1 (33.3)	0 (0)
	Vancomycin	6	4	1 (25)	0 (0)	1 (25)	2 (50)	2	0 (0)	0 (0)	0 (0)	2 (100)
	Imipenem/cilastatin	8	8	0 (0)	1 (12.5)	1 (12.5)	6 (75)	0	0 (0)	0 (0)	0 (0)	0 (0)
	Ceftriaxone	13	8	3 (37.5)	2 (25)	2 (25)	1 (12.5)	5	1 (20)	2 (40)	1 (20)	1 (20)
	Piperacillin/tazobactam	11	8	1 (12.5)	1 (12.5)	2 (25)	4 (50)	3	0 (0)	0 (0)	1 (33.3)	2 (66.7)
Reserve	Linezolid	0	NP	NP	NP	NP	NP	NP	NP	NP	NP	NP
	Colistin	4	4	2 (50)	0 (0)	1 (25)	1 (25)	0	0 (0)	0 (0)	0 (0)	0 (0)

N_1_ = prescriptions where gram-negative (sensitivity) was checked; N_2_ = prescriptions where gram-negative (resistance) was checked. *n* = total prescriptions where gram-negative (sensitivity) and gram-negative (resistance) were checked; NP = not performed.

**Table 7:** Multinomial logistic regression analyses of factors associated with AWaRe classification and the usage pattern of antibiotics.

Total = 768 (100%)	Access 244 (31.8%)			Watch 457 (59.5%)			Reserve 67 (8.7%)		
Characteristics	β	AOR (95% CI)	*p*-Value	β	AOR (95% CI)	*p*-Value	β	AOR (95% CI)	*p*-Value
**Gender**									
Male	Referent			Referent			Referent		
Female	0.34	0.267 (0.078–1.298)	0.448	−0.13	0.185 (0.041–0.977)	0.058	0.16	0.356 (0.298–1.718)	0.103
**Length of stay (days)**									
1	Referent			Referent			Referent		
2–3	0.21	0.218 (0.074–0.778)	0.116	−0.24	0.193 (0.048–0.387)	0.063	0.17	0.197 (0.067–0.989)	0.035
4–5	0.32	0.378 (0.055–1.927)	0.238	0.23	0.378 (0.055–1.927)	0.082	0.28	0.276 (0.359–2.174)	0.029
6–7	−0.12	0.514 (0.298–1.718)	0.137	−0.23	0.876 (0.359–2.174)	0.096	0.19	0.569 (0.144–0.891)	0.047
>7	−0.18	0.389 (0.117–1.498)	0.143	−0.36	0.444 (0.363–0.875)	0.059	0.15	0.914 (0.298–1.718)	0.041
**Number of antibiotics given per day**									
1	Referent			Referent			Referent		
2	0.12	0.391 (0.192–0.767)	0.319	0.11	1.091 (0.581–2.561)	0.278	0.05	0.219 (0.192–0.767)	0.014
3	−0.14	0.485 (0.185–1.317)	0.174	−0.09	0.485 (0.185–1.317)	0.094	0.08	0.787 (0.178–0.956)	0.032
≥4	−0.16	0.289 (0.078–0.456)	0.152	−0.12	0.671 (0.612–1.781)	0.198	0.07	1.293 (0.185–0.478)	0.048
**Route of administration**									
Parenteral (intravenous)	Referent			Referent			Referent		
Oral	0.25	0.478 (0.362–0.991)	0.231	0.26	1.091 (0.581–2.561)	0.173	0.22	0.719 (0.451–1.981)	0.155

AOR = Adjusted Odds ratio.

## Data Availability

Data are available upon a reasonable request.
